# Ferritin-mediated neutrophil extracellular traps formation and cytokine storm via macrophage scavenger receptor in sepsis-associated lung injury

**DOI:** 10.1186/s12964-023-01440-6

**Published:** 2024-02-02

**Authors:** Hao Zhang, Dan Wu, Yanghanzhao Wang, Yuxin Shi, Yuwen Shao, Fu Zeng, Charles B. Spencer, Lilibeth Ortoga, Dehua Wu, Changhong Miao

**Affiliations:** 1 Department of Anesthesiology, Zhongshan Hospital, Fudan University, 180# Feng-Lin Road, Shanghai, 200032 China; 2 Shanghai Key Laboratory of Perioperative Stress and Protection, Shanghai, China; 3 Department of Anesthesiology, Shanghai Medical College, Fudan University, Shanghai, China; 4 Department of Cardiac surgery, The Ohio State University, Columbus, USA; 5 Department of Biomedical Engineering, The Ohio State University, Columbus, USA; 6 Department of Anesthesiology, Shanghai Songjiang District Central Hospital, Shanghai, China

**Keywords:** Ferritin, Neutrophil extracellular traps, sepsis-associated acute lung injury, Macrophage scavenger receptor

## Abstract

**Background:**

Sepsis is a severe systemic inflammatory disorder manifested by a dysregulated immune response to infection and multi-organ failure. Numerous studies have shown that elevated ferritin levels exist as an essential feature during sepsis and are able to suggest patients’ prognoses. At the same time, the specific mechanism of ferritin-induced inflammatory injury remains unclear.

**Methods:**

Hyper-ferritin state during inflammation was performed by injecting ferritin into a mouse model and demonstrated that injection of ferritin could induce a systemic inflammatory response and increase neutrophil extracellular trap (NET) formation.Padi4^−/−^, Elane^−/−^ and Cybb^−/−^ mice were used for the NETs formation experiment. Western blot, immunofluorescence, ELISA, and flow cytometry examined the changes in NETs, inflammation, and related signaling pathways.

**Results:**

Ferritin induces NET formation in a peptidylarginine deiminase 4 (PAD4), neutrophil elastase (NE), and reactive oxygen species (ROS)-dependent manner, thereby exacerbating the inflammatory response. Mechanistically, ferritin induces the expression of neutrophil macrophage scavenger receptor (MSR), which promotes the formation of NETs. Clinically, high levels of ferritin in patients with severe sepsis correlate with NETs-mediated cytokines storm and are proportional to the severity of sepsis-induced lung injury.

**Conclusions:**

In conclusion, we demonstrated that hyper-ferritin can induce systemic inflammation and increase NET formation in an MSR-dependent manner. This process relies on PAD4, NE, and ROS, further aggravating acute lung injury. In the clinic, high serum ferritin levels are associated with elevated NETs and worse lung injury, which suggests a poor prognosis for patients with sepsis. Our study indicated that targeting NETs or MSR could be a potential treatment to alleviate lung damage and systemic inflammation during sepsis.

Video Abstract

**Supplementary Information:**

The online version contains supplementary material available at 10.1186/s12964-023-01440-6.

## Introduction

Ferritin was previously thought to serve as an acute-phase response protein [1]. Recent studies have unequivocally demonstrated the critical role it plays in a multitude of conditions, including infections, autoimmune diseases, and multiple organ injuries [2]. Elevated ferritin in patients with conditions of bacterial or viral infections is a prevalent phenomenon [3]. The investigators found an upregulation of ferritin levels present in patients with sepsis-induced acute organ dysfunction, including acute kidney injury and acute liver injury [4].

Sepsis is defined as a life-threatening disorder of the host’s response to infection. The presence of severe sepsis often results in systemic multi-organ dysfunction, with sepsis-associated acute lung injury emerging as a significant contributor to unfavorable patient outcomes [5]. During the process of sepsis, the infection triggers a substantial release of pro-inflammatory mediators, which subsequently activate neutrophils and result in significant damage to tissue and organ function [6]. Sepsis-induced acute lung injury (SI-ALI) is one of the most common complications of sepsis, the further development of which is acute respiratory distress syndrome, leading to severe respiratory insufficiency and a poor prognosis for clinical patients [7]. Recent studies have found a significant up-regulation of serum ferritin levels in patients with severe COVID-19-induced infections and lung injury [8]. The poor prognosis of patients was positively correlated with patients’ serum ferritin levels [9]. Available evidence suggests that intracellular iron homeostasis is important in the development of acute lung injury. Among them, processes such as ferritinophagy and ferroptosis, which have garnered significant attention in recent years, have been demonstrated to exert an influence on sepsis-associated acute lung injury through a multitude of mechanisms [10, 11]. However, it is still unclear whether extracellular ferritin levels directly cause lung inflammation and the specific mechanisms involved.

A large number of studies have shown the critical role of neutrophils in sepsis and SI-ALI [12]. Neutrophil extracellular traps (NETs) are a form of reticular material released by neutrophils in response to stimulation during their involvement in the immune-inflammatory response [13]. In the pathological process of SI-ALI, the formation of large numbers of NETs is associated with excessive inflammatory responses and lung tissue damage [12, 14, 15]. The precise mechanism underlying the induction of SI-ALI by NETs remains incompletely understood at present.

Macrophage scavenger receptor (MSR), also known as CD204, plays a key function in a variety of pathophysiological processes, including inflammation [16]. Yu et al. reported that scavenger receptors, an important superfamily of membrane-bound receptors, function as ferritin receptors, and play an important role in maintaining iron homeostasis in vivo. Among them, Msr1 was shown to be involved in the internalization of ferritin [17]. Recent studies have shown that Msr1 is involved in the generation of NETs during hepatic inflammatory processes [18].

Herein, we demonstrate that sepsis patients with high ferritin levels have a systemic hyperinflammatory state. Ferritin levels are proportional to the severity of sepsis-associated lung injury. Our study delved further into ferritin’s role in sepsis-associated acute lung injury by administering intraperitoneal injections of ferritin to simulate the hyper-ferritin state during sepsis. We discovered that ferritin triggers the formation of NETs by activating scavenger receptor Msr in neutrophils. This process is dependent on peptidyl arginine deiminase 4 (PAD4), neutrophil elastase (NE), and reactive oxygen species (ROS). Our study elucidates a novel mechanism by which ferritin exacerbates lung injury via elevated NET production. This study provides potential ideas for the clinical treatment of patients with sepsis-associated lung injury characterized by high ferritin.

## Materials and methods

### Ethnic statements and patients

The Ethics Committee of Zhongshan Hospital, Fudan University (B2021-182R) approved the collection of blood samples from both healthy donors and septic patients. All participants or their relatives provided written informed consent. For septic patients, 10 millilitres of peripheral venous blood were collected within 1 h of their admission to the Intensive Care Unit (ICU). The hospitalization outcomes of these patients were recorded, with those who did not survive being classified as “dead” and those who did survive as “alive.” Mouse experiments were conducted following institutional protocols approved by the animal review committee of Zhongshan Hospital, Fudan University.

### Animals

Female, 8–12 week old WT FVB/n (#215) and C57BL/6 (#219) mice were purchased from the Shanghai Model Organisms Center. Msr1-deficient (Msr1−/−) mice in the C57BL/6 background were obtained from Nanjing Medical University (Nanjing, China) and purchased from The Jackson Laboratory (#006096, RRID: IMSR_JAX: 006096).Padi4^−/−^, Elane^−/−^ and Cybb^−/−^ mice were obtained from Shanghai Model Biology (Padi4^−/−^: #NM-KO-190334, Elane^−/−^: #NM-KO- 201544, Cybb^−/−^: #NM-KO-18031). Animals were housed in pathogen-free conditions, with no more than six animals per cage, in a 12-hour light/dark cycle with free access to rat food and water, at an ambient temperature of 22–24 °C and 50–70% humidity. All experiments were conducted on sex- and age-matched animals between 8 and 10 weeks old. Ferritin (F4503, sterile filtered, Sigma-Aldrich, USA) was injected intraperitoneally (i.p.) at a dose of 60 μg/g body weight. After 3, 6, 12 and 24 h of treatment, mice were anaesthetized by inhalation of isoflurane to measure body weight and blood was collected, euthanized with 2.5% chloral hydrate (0.1 mL/10 g), and the lungs were removed by rapid dislocation. The lungs were weighed. Blood and serum were collected for flow cytometry and cytokine analysis. Lung specimens were collected, fixed in 4% paraformaldehyde, and paraffin-embedded. Additional lung samples were stored at − 80 °C.

Anti-Ly6G antibody (500 μg/mouse, ab238132, Abcam, Cambridge, MA, USA) or rat IgG2a isotype control (BE0089, BioXcell) was given 24 h and 2 hours before ferritin injection. The following drugs were injected intraperitoneally: CI-amidine (50 mg/kg, Sigma-Aldrich, St Louis, MO, USA), DPI (1 mg/kg, D2926, Sigma-Aldrich, USA), sivelestat (50 mg/kg, HY-17443, MCE, China).

Before bone marrow transplantation (BMT), 8-week-old female C57BL/6 wild-type mice were lethally irradiated with 10 Gy using a caesium gamma source. Then, 5 × 10^6^ cells of donor bone marrow obtained from female donor mice aged 8–12 weeks old, including WT, Padi4^−/−^, Elane^−/−^, and Cybb^−/−^, were intravenously injected into the recipient mice after irradiation. After 4 weeks of BMT, the mice were injected intraperitoneally with 60 μg/g ferritin.

### Isolation and stimulation of neutrophils

Neutrophils were isolated using the Human and Mouse Neutrophil Isolation and Purification Kit (TBD Sciences, Tianjin, China) for subsequent experiments. Briefly, neutrophils were isolated from human or mouse blood samples by stratification using the Neutrophil Isolation Reagent and centrifuged at 800 g for 30 min at room temperature. The lower band containing neutrophils was carefully aspirated and washed with PBS after removing erythrocytes with erythrocyte lysate. The remaining cells were resuspended with 1640 (Gibco, US) containing 10% fetal bovine serum (FBS) (Gibco). The isolated neutrophils were then inoculated in 6-well plates (2 × 10^6^ cells per well). Neutrophils were stimulated in vitro with 50 nM PMA (MKBio, Shanghai, China) or ferritin (10–1000 nM, F4503, Sigma-Aldrich) for 4 hours.

### Lung wet-to-dry ratio

To calculate the lung wet-to-dry ratio, the left lung tissues of mice were collected and weighed while wet. The surface water was then absorbed and the tissues were dried at 70 °C for 48 hours to obtain the dry weight. The wet/dry weight ratio was then calculated by dividing the wet weight by the dry weight.

### Quantification of ds-DNA, MPO-DNA complexes and multi-inflammatory factors

DNA in human and mouse plasma was quantified following the manufacturer’s instructions and using the kits for Quant-iTTM PicoGreen® dsDNA Reagent (Invitrogen, MA, USA). The concentration of MPO-DNA complexes in human and mouse serum was determined using a capture ELISA kit. (ab119605, Abcam). In detail, wash the neutrophil or NET dish first, isolate neutrophils collect ex vivo NET structures by mechanical agitation and then apply MPO-DNA complex in the collected material. To analyze MPO-DNA, 96-well ELISA Plates were coated with 5 mg/ml anti-MPO capture antibody overnight at 4 °C. After three washes in PBS, the wells were blocked with 5% BSA in PBS for 45 min at room temperature. Then, 50 ml of patient serum together with peroxidase-labeled anti-DNA monoclonal antibodies were added and incubated for 2 h at room temperature, and the plates were washed three times with washing buffer, After incubation at 37 °C for 1 h, the signals were measured at 405 nm. Inflammatory factors were measured separately using specific ELISA kits: TNF-α(abs520010, Absin), IL-6 (abs552805, Absin), IL-1β (abs520001, Absin), MCP-1(abs520016, Absin), and IL-10 (abs520005, Absin).

### Immunofluorescence

Cells were fixed with 4% formaldehyde (Servicebio, Wuhan, China) and blocked with 1% BSA (Biosharp, Hefei, China), and incubated with primary antibodies against CitH3 (1:100, ab5103, Abcam) and MPO (1:50, AF3667, R&D Systems) at 4 °C overnight. The same primary antibodies were used for paraffin-embedded lung tissue sections. These sections were first deparaffinized and rehydrated, blocked in 1% BSA, and then incubated overnight at 4 °C. The next day, a fluorescent secondary antibody was added to both the cells and slides and left at room temperature for 1 h. The nuclei were then stained with DAPI and observed under a microscope (Olympus, Tokyo, Japan).

### Scanning electron microscopy

Neutrophils or lung tissue samples were first fixed in 2.5% glutaraldehyde and postfixed with 1% osmic acid for 2 h. Gradual dehydration was then carried out using a series of ethanol concentrations: 30, 50, 70, 80, 90, and 100%. Next, the neutrophils were dried and coated with gold before being imaged using a scanning electron microscope (SU8100, Hitachi, Tokyo, Japan). As for the lung tissue samples, they were embedded in 812 resin and stained with 2% uranyl acetate. Ultrastructural images of mitochondria were taken using a transmission electron microscope (HT7700, Hitachi).

### Histopathological analysis

To assess the extent of lung injury, tissue sections that were embedded in paraffin were treated with xylene and graded ethanol for hydration before being stained with hematoxylin and eosin. A scoring system graded hemorrhage, alveolar oedema, thickening of alveolar septa, and leukocyte infiltration. The scores were added to determine the lung injury score, ranging from 0 to 3 (0 = normal; 1 = mild; 2 = moderate; 3 = severe).

### Immunohistochemistry

Tissue sections were deparaffinized, hydrated, and blocked with 5% BSA. Primary antibodies, including anti-citH3 (1:200, ab5103, Abcam), anti-NE (1:50, sc-55,549, Santa Cruz) and anti-MPO (1:200, AF3667, R&D). were applied and left at 4 °C overnight. Secondary antibodies with horseradish peroxidase (HRP) were used, followed by staining and observation under light microscopy. We examined the slides using a Carl Zeiss light microscope (Jena, Germany).

### Masson’s trichrome staining

Paraffin-embedded tissue sections were dewaxed, hydrated, and stained with Masson’s trichrome (Servicebio). Sections were then sealed with neutral resin and observed under a light microscope (Carl Zeiss).

### RNA sequencing

Total RNA was extracted using TRIzol reagent (Invitrogen) and we assessed the concentration and integrity of RNA for further RNA library construction. RNA libraries were created using the KAPA Stranded RNA- seq Library Preparation Kit (Illumina) and sequenced using the Illumina NovaSeq 6000 platform. We used the DESeq2 package in R (R 4.2.1) to perform differential expression analysis on the sequencing data, and defined genes with *P* < 0.05 as differentially expressed genes (DEGs).

### Real-time quantitative PCR (RT-qPCR)

To obtain RNA from lung tissues or neutrophils, we used TRIzol reagent (Invitrogen) and then reverse-transcribed it into cDNA with a PrimeScript RT reagent kit (Takara, Shinga, Japan). We used a TB Green PCR kit (Takara) for RT-qPCR and measured relative mRNA levels with β-actin as the reference gene.

### Western blotting

RIPA lysis buffer (Solarbio, Beijing, China) was used to extract total proteins. After separation through sodium dodecyl sulfate-polyacrylamide gel electrophoresis (SDS-PAGE), the proteins were transferred onto polyvinylidene fluoride (PVDF) membranes. These membranes were blocked with 5% milk and then exposed to primary antibodies at 4 °C overnight, followed by HRP-conjugated secondary antibodies. Detection of signals was carried out using an ECL chemiluminescence kit (Tanon, Shanghai, China). The following antibodies were used in this experiment: anti-NE (1:1000, ab68672, Abcam), anti-citH3 (1:1000, ab5103, Abcam), and anti-GAPDH antibody (1:2000, 5174, Cell Signaling Technology).

### Flow cytometry

Staining of digested single cell suspensions of lung tissue and blood leukocytes was performed for 15 minutes using fluorescently labelled antibodies: PerCP rat anti-mouse CD45 (30-F11 clone, 1:100, 557,235, BD), FITC anti-mouse CD11b (M1/70 clone, 1:100, 101,206, Biolegend), PE-Cy™7 rat anti-mouse Ly6G (clone 1A8, 1:100, 560,601, BD), APC anti-mouse Ly6C (clone HK1.4, 1:100, 560,595, BD), FITC rat anti-mouse CD3 (clone 17A2, 1:100, 561,798, BD), PE-Cy™7 rat anti-mouse CD4 (clones RM4–5 clone, 1:100, 552,775, BD), APC-H7 rat anti-mouse CD8a (clone 53–6.7, 1:100, 560,182, BD), APC rat anti-mouse CD19 (clone 1D3, 1:1000, 550,992, BD), PE rat anti-mouse CD49b (clone DX5, 1:1000, 553,858, BD), PE anti-mouse F4/ 80 (BM8 clone, 1:1000,123,110, Biolegend) and Alexa Fluor 647-labelled anti-mouse Msr1 (2F8 clone, 1:1000, MCA1322A647, AbD Serotec, NC, USA). Staining was performed with PE anti-human CD11b (ICRF44 clone, 1:1000, 555,388, BD), PE-Cy7 anti-human CD66b (G10F5 clone, 1:1000, 305,116, Biolegend) and APC anti-human Msr1 (7C9C20 clone, 1:1000, 371,905, Biolegend) stained human blood. All assays were performed by FACS Canto II cytometer (BD). Cell sorting was performed using FACSAria. Data were analysed using FlowJo software (Tree Star, Inc., Ashland, OR).

### Statistic analysis

All data were statistically analyzed using SPSS 20.0 software (Chicago, IL, USA) and GraphPad Prism v8.0 software. Quantitative data were expressed as mean ± SEM (standard error of the mean) or mean ± SD (standard deviation). Gaussian data were analyzed by unpaired two-sided t-test, one-way ANOVA or two-way ANOVA (χ2 test), whereas nonparametric data were analyzed using the Mann-Whit U test or Wilcoxon rank sum test for assessment. Bonferroni post hoc tests were used to compare all paired treatment groups. All tests were two-sided and a *p*-value of < 0.05 was considered statistically significant.

## Result

### Ferritin induces acute lung injury in vivo and promotes the accumulation of neutrophils

**Fig. 1:**
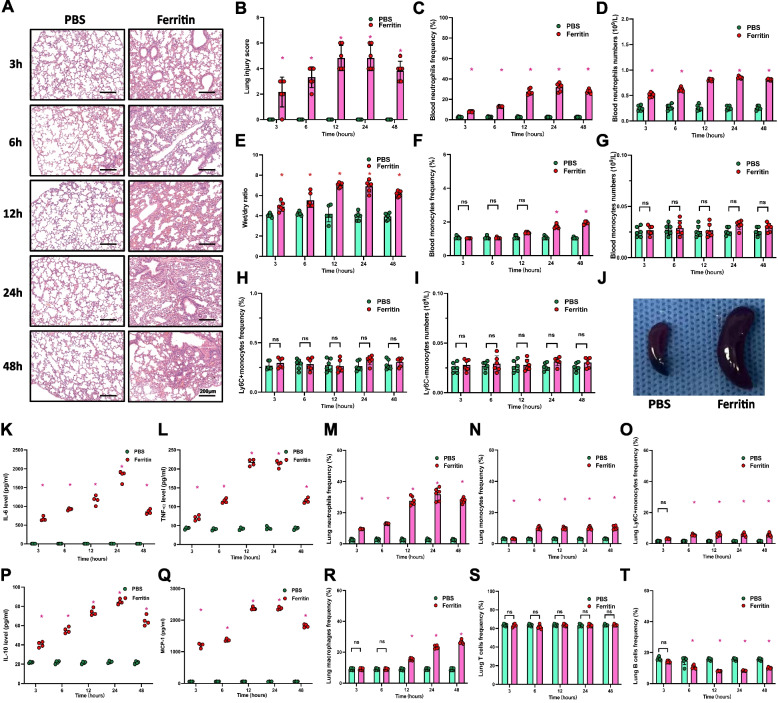
Ferritin induces systemic inflammation and lung injury in vivo, resulting in lung neutrophil accumulation. Mice were treated with ferritin injections for the corresponding times (3 h, 6 h, 12 h, 24 h, 48 h). **A** Lung tissues’ HE staining of ferritin-injected mice. **B** Lung injury scores of ferritin-treated mice (*n* = 6). **C** and **D** Neutrophils frequency and cell number in peripheral blood of mice (*n* = 6). **E** Lung wet to dry rate (*n* = 6). **F** and **G** Frequency and number of peripheral blood monocytes. **H** and **I** Frequency and number of Ly6C+ monocytes in peripheral blood (*n* = 6). **J** Splenic appearance of mice 12 h after ferritin treatment. **K**, **L**, **P**, and **Q** Plasma inflammatory factor levels in mice (IL-6, TNF-α, IL-1β, and MCP-1, *n* = 4). **M**, **N**, **O**, **R**, **S**, and **T** Lung tissue neutrophil, monocyte, Ly6C+ monocyte, macrophage, T-cell, and B-cell frequencies (*n* = 6) *p < 0.05

To investigate the pathological role of ferritin in sepsis-induced acute inflammatory lung injury, we simulated the elevated ferritin during sepsis by intraperitoneal injection of ferritin. After 3 h of intraperitoneal ferritin injection, we observed a significant upregulation of lung injury (Fig. 1A, B). This injury showed a time-dependent pattern that peaked at 12 h post-injection and persisted until 24 h. Meanwhile, ferritin injection increased the number and frequency of peripheral blood neutrophils (Fig. 1C, D). We further assessed the extent of lung injury using lung dry/wet rate, and the results were consistent with the lung injury score (Fig. 1E) and subsequently, we observed a mild increase in the number of monocytes in the peripheral blood (Fig. 1F, G). However, Ly6C+ cells did not produce significant changes (Fig. 1H, I). To further determine the effect of ferritin on systemic inflammatory responses in mice, serum levels of proinflammatory cytokines, including interleukin (IL) -6, tumour necrosis factor (TNF)-α, IL-10 and Monocyte chemoattractant protein (MCP)-1 were measured in mice treated with ferritin (Fig. 1K, L, P and Q). IL-10, MCP-1, IL-6, and IFN-γ were all elevated at 3 h after ferritin injection. We also observed significant enlargement of the spleen in mice 6 h after ferritin injection (Fig. 1J). Localized immune cell infiltration can indicate the severity of tissue damage during acute inflammation. We utilized flow cytometry to measure the occurrence of inflammatory cells in lung tissue following ferritin injection (Fig. 1M-O, R-T). Consistent with the results of serum analyses, there was a significant accumulation of neutrophils in the lungs within 3 hours of receiving a ferritin injection. This trend continued to escalate and peaked at the 24-hour mark after injection (Fig. 1M). Monocytes and Ly6C+ cells in lung tissue also increased after ferritin injection (Fig. 1N, Q). Further analysis showed that macrophages increased 12 h after ferritin injection in lung tissue of mice injected with ferritin compared to the control group, while T cells did not show statistically significant changes, and B cells showed a downregulation at 6 h after injection (Fig. 1R-T). To summarize, the data indicates that ferritin contributes to both systemic and lung inflammatory injury. This is primarily demonstrated by an increase in circulating neutrophils, enlargement of the spleen, and an elevation in acute lung injury markers. Additionally, ferritin can cause an accumulation of neutrophils in the lungs during acute lung injury.

### Ferritin induces the generation of NETs

**Fig. 2:**
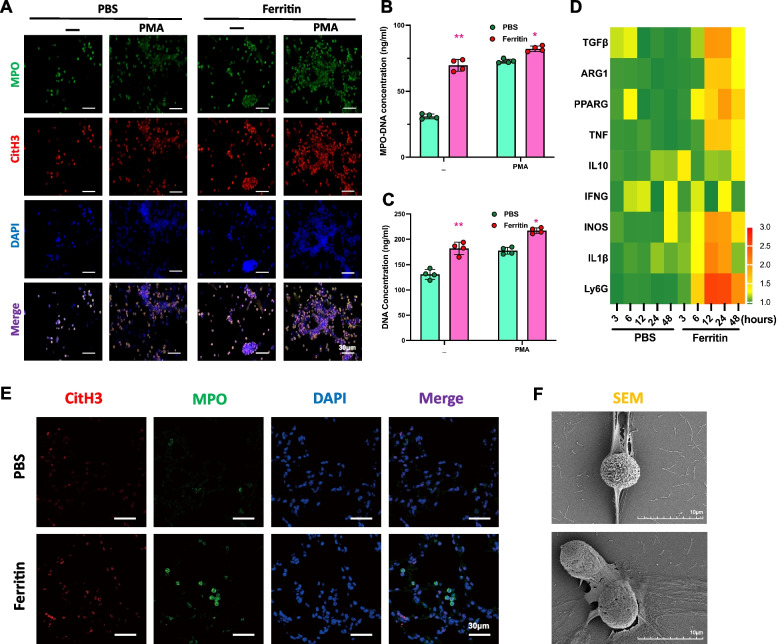
Ferritin promotes the formation of NETs. Neutrophils isolated from ferritin-treated and control mice were treated with PMA. **A** Representative immunofluorescence staining of spontaneous, PMA-induced NET formation in ferritin-treated mice at 12 h BMDNs. Neutrophils were stained with MPO (green), CitH3 (red) antibodies and DAPI (blue). Scale bar, 30 μm. **B** Serum MPO-DNA complexes and (**C**) dsDNA concentration in cell culture supernatant. **D** Heatmap of RNA-seq differential analysis of BMDNs in PBS and ferritin-injected mice. **E** Immunofluorescence staining images of mice lung tissue after PBS or ferritin treatment. (MPO, green; CitH3, red; DAPI, blue. Bar = 30 μm). **F** Image of SEM (scanning electron microscopy) showed the morphology of extracellular meshes NETs formation after ferritin injection. (bar = 10 μm) *p < 0.05

NETs released by neutrophils play a vital role in acute inflammation. Having observed the inflammatory effects of injecting ferritin and the localized accumulation of neutrophils in mice’s bodies and lungs, it is meaningful that we investigate how this stimulus affects the formation of NETs. We isolated and extracted neutrophils from the bone marrow of mice injected with ferritin for 24 h and stimulated them with PMA. We performed immunofluorescence (IF) using myeloperoxidase (MPO) and citrullinated histone H3 (CitH3), two recognized biomarkers of NETs formation (Fig. 2A). IF results showed that BMDNS from ferritin-treated mice showed an increased ability to release NETs. Corresponding with this observation, DNA concentration and MPO-DNA concentration were further measured via ELISA (Fig. 2B, C). These results indicated a contributing role of ferritin to neutrophils NETosis.

We observed that ferritin promotes neutrophil recruitment to lung tissue and leads to acute lung injury (Fig. 1). RNA-seq results of lung tissues from ferritin-treated mice showed that inflammation-related genes appeared to be up-regulated in a time-dependent manner after ferritin injection, and neutrophil-specific expression of Ly6G appeared to be markedly elevated at 6 h after ferritin treatment and reached a maximum value at 12 h (Fig. 2D). To determine the production of NETs in the lungs, we selected the lung tissues of a mouse model for immunofluorescence staining at 12 h after ferritin injection, and the results showed that ferritin injection induced a significant increase in the production of NETs in the lung tissues (Fig. 2E). We extracted peripheral blood leukocytes from mice 12 h after ferritin injection and observed the neutrophils using scanning electron microscopy (SEM). The morphology of ferritin-induced peripheral blood NET production could be observed (Fig. 2F). Taken together, these results suggest that ferritin exacerbates NETs production in addition to inducing lung tissue recruitment of neutrophils.

### Neutrophils are necessary for ferritin-induced acute lung injury

**Fig. 3:**
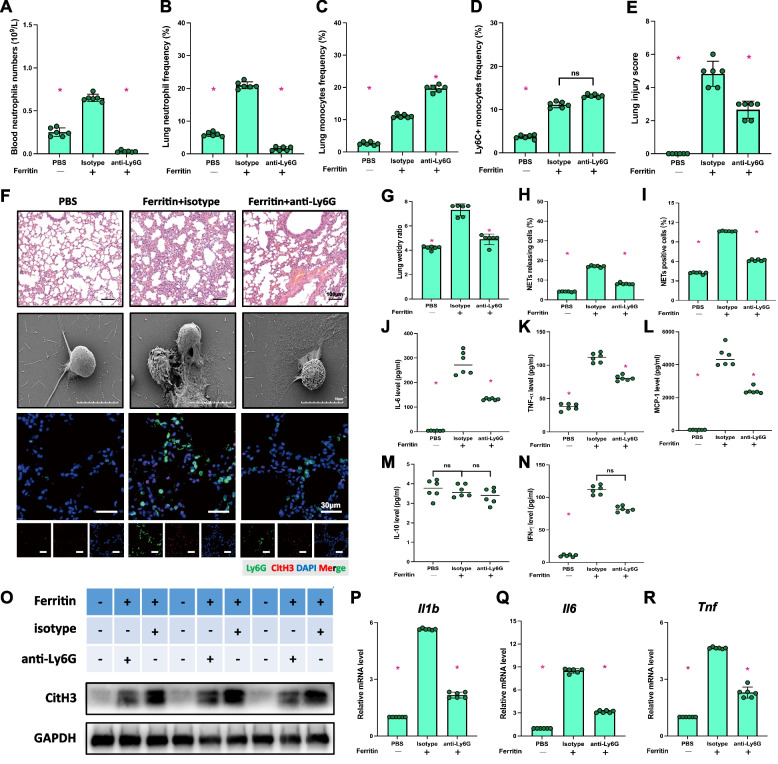
Ferritin-induced systemic inflammatory response and lung injury were dependent on neutrophils. Co-administration of anti-Ly6G antibody to ferritin-treated mice for 12 h. **A**, **B**, **C** and **D** Neutrophil number, frequency, monocyte frequency and Ly6C+ neutrophil frequency in mouse lungs. **E** Lung injury score. **F** HE staining of 12 h mouse lung tissue (Scale bar = 100 μm), SEM images of BMDN neutrophil NETs (Scale bar = 10 μm), and IF images of lung tissue (Ly6G, green; CitH3, red; DAPI, blue. Scale bar = 30 μm). **G** lung wet to dry rate. **H** The proportion of cells releasing NETs in peripheral blood. **I** The proportion of NETs-positive cells in lung tissue. **J**, **K**, **L**, **M** and **N** Mouse plasma levels of inflammatory factors: IL-6, TNF-α, MCP-1, IL-10 and IFN-γ. **O** Immunoblot of mouse lung tissue for CitH3 protein expression. **P**, **Q** and **R** Il1b, Il6, Tnf mRNA levels in lung tissue. *p < 0.05

We noticed a noteworthy rise in neutrophils in mice’s circulating and lung tissues following the ferritin treatment. Next, we wanted to investigate whether neutrophils play a decisive role in ferritin-induced lung injury. Mice showed a peak in systemic inflammation levels 12 h after ferritin injection and exhibited the most significant lung injury, so we chose these mice as a model for subsequent experiments (Fig. 1A). The use of anti-Ly6G antibody was employed to eliminate neutrophils. It was observed that neutrophils’ number and frequency in both peripheral blood and mouse lung tissues were significantly reduced after ferritin injection, as per the flow cytometry results (Fig. 3A, B). Meanwhile, the anti-Ly6G antibody could cause an increase in the frequency of monocytes in lung tissues (Fig. 3C), and had no significant effect on Ly6C+ monocytes (Fig. 3D). HE staining, lung dry and wet rate, and lung tissue damage scores showed that the use of anti-Ly6G antibody significantly reversed ferritin-induced lung acute injury (Fig. 3E-G). In addition, WB results showed reduced citH3 expression in the lung with neutrophil depletion (Fig. 3O). The findings from the IF and SEM tests indicate that administering anti-Ly6G antibodies successfully inhibited the formation of NETs in the lungs of mice upon ferritin stimulation (Fig. 3F). Moreover, the use of anti-Ly6G antibodies significantly reduced the number of NET-positive cells in the peripheral blood of mice, as illustrated in Fig. 3H-I. We further measured the levels of inflammatory factors in mouse serum using ELISA to investigate the effect of neutrophil depletion on systemic inflammation in ferritin-treated mice. The ferritin-induced up-regulation of IL-6, TNF-α and MCP-1 levels in serum was significantly reversed after the use of ly6g antibody (Fig. 3J-L), but there were no statistically significant differences observed in IL-10 and IFN-γ (Fig. 3M, N). Meanwhile, inhibition of neutrophil reduced Il1b, Il6 and TnfαmRNA levels in the lung tissue. The above observations suggest that neutrophils play a key role in ferritin-triggered systemic inflammation and acute lung injury.

### PAD4, NE and ROS are required for ferritin-induced NETosis

**Fig. 4:**
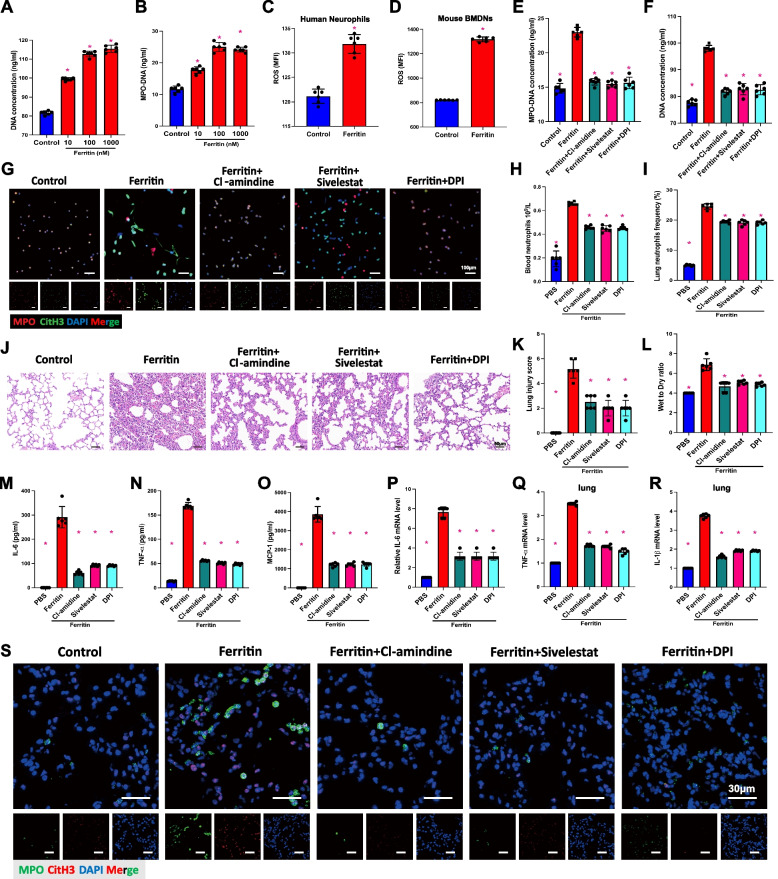
PAD4, NE and ROS are required for the formation of NETs induced by ferritin. Ferritin-injected mice were treated with PAD4, NE, and ROS inhibitors. **A** DNA concentration and (**B**) MPO-DNA complex were measured in cell culture supernatants after exposing healthy human neutrophils to indicated concentrations of ferritin. **C** ROS levels in healthy human neutrophils after ferritin stimulation for 12 h in vitro. **D** ROS levels in mouse BMDNs after 12 h of ferritin stimulation. **E** and **F** the concentration of MPO-DNA in human neutrophils’ culture supernatants was measured after being stimulated with ferritin alone or in combination with Cl-amidine, sivelestat, and DPI. **G** IF images of the effect of ferritin alone and combined with inhibitors on the formation of neutrophil NETs. (MPO, red; CitH3, green; DAPI, blue. Bar = 100 μm). **H** and **I** Neutrophil counts and frequencies in peripheral blood and lung tissue of mice after application of ferritin alone and in combination with various inhibitors. **J** HE staining of mouse lung tissue (Scale bar = 50 μm). **K** and **L** Lung injury score and wet-to-dry ratio. **M**, **N** and **O** Plasma concentrations of inflammatory factors IL-6, TNF-α and MCP-1 in mice. **P**, **Q** and **R** Lung tissue mRNA levels of inflammatory factors including Il-6, Tnf and Il-b. **S** IF-stained images of NETs markers in lung tissue. (MPO, green; CitH3, red; DAPI, blue. Bar = 30 μm)

To further investigate the specific mechanism by which ferritin promotes neutrophil NETs production, we isolated and extracted neutrophils from the peripheral blood of healthy donors for in vitro ferritin stimulation. We treated neutrophils with 10 ~ 1000 nM ferritin to select the optimal stimulation concentration for the in vitro model and found that ferritin significantly stimulated the production of neutrophil NETs and showed the best effect at a concentration of 100 nM (Fig. 4A, B). In addition, intracellular ROS levels increased in both ferritin-treated human neutrophils and ferritin-treated mouse BMDNs for 6 h (Fig. 4C, D).

A large body of evidence suggests that ROS, PAD4 and NE play important roles in the generation of NETs. We hypothesize that the increase in neutrophil NET production caused by ferritin could be due to its impact on these key factors. Next, we pretreated neutrophils with Cl-amidine (PAD4 inhibitor), Sivelestat (NE inhibitor), or DPI (NADPH oxidase inhibitor), respectively, and then combined them with 100 nM ferritin for stimulation. After 12 h of ferritin stimulation, we extracted neutrophil culture supernatants for the determination of cell-free DNA, MPO-DNA. The results showed that all inhibitors inhibited ferritin-induced NET formation (Fig. 4E, F). IF staining also showed that combining three inhibitors separately inhibited ferritin-induced NET generation (Fig. 4G). The above results tentatively suggest that PAD4, NE, and ROS play indispensable roles in ferritin-induced NET formation.

We then verified the effects of the above inhibitors on ferritin-induced acute lung injury in mice. Flow cytometry results showed that Cl-amidine, Sivelestat and DPI significantly reduced the number of neutrophils in circulating and lung tissues (Fig. 4H, I). HE results showed that the combined inhibitor significantly ameliorated ferritin injection-induced inflammatory infiltration and tissue damage in lung tissue (Fig. 4J). This result was also verified in the lung injury score and lung wet-to-dry rate (Fig. 4K, L). In addition, we found that inhibition of PAD4, NE and ROS ameliorated the systemic and local inflammatory conditions in the lungs of mice. Pretreatment with Cl-amidin, Sivelestat or DPI reduced the serum levels of IL-6, TNF- α, and MCP-1 in ferritin-injected mice (Fig. 4M-O), and at the same time, decreased the levels of Il1b, Il6, and MCP-1 in lung tissues (Fig. 4P-Q).

**Fig. 5:**
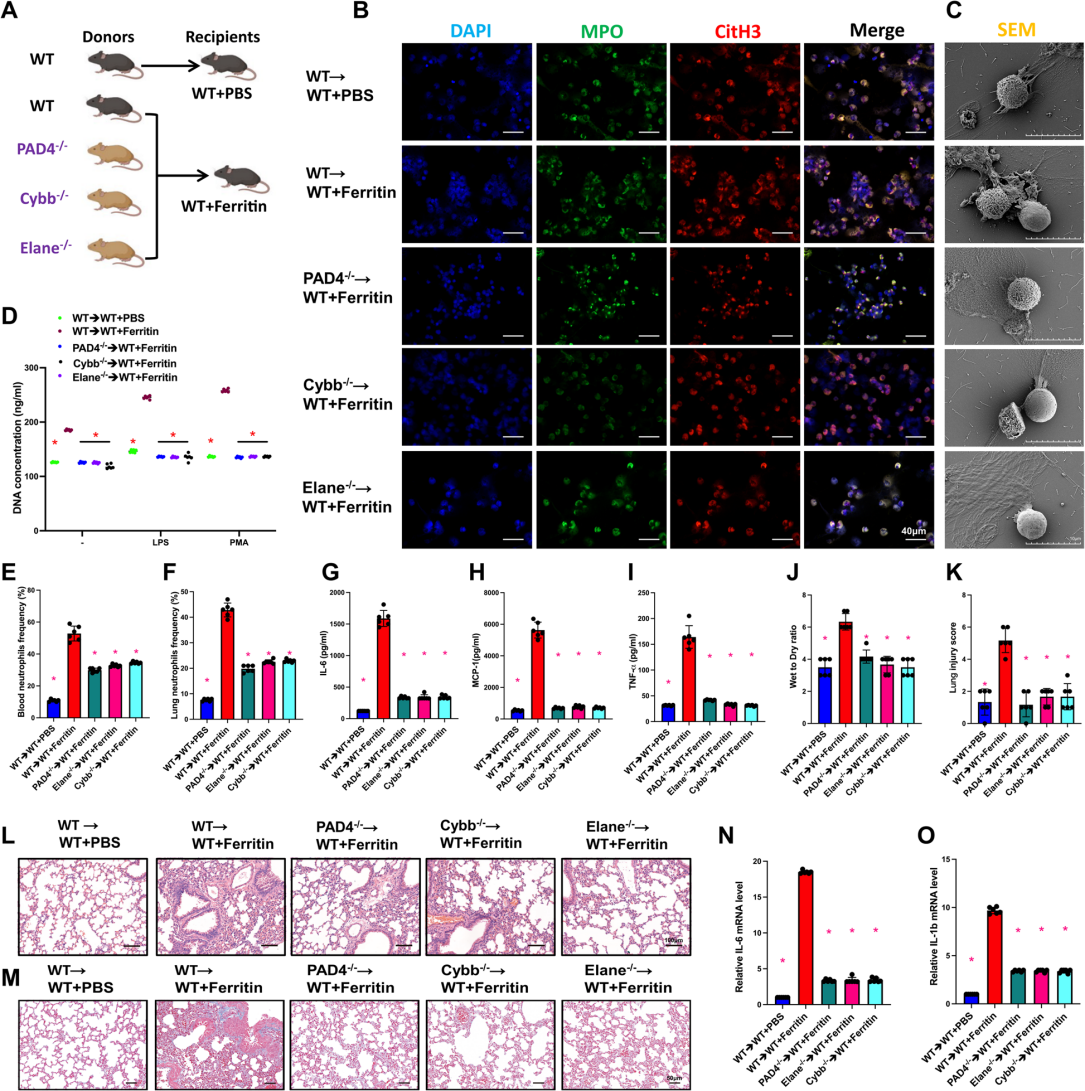
Bone marrow of donors with PAD4, NE, ROS gene defects inhibited ferritin-induced NET production in WT recipient. **A** Overview of the experiment: WT, Padi4−/−, Elane−/−, and Cybb−/− mice had their bone marrow transplanted into WT recipients. The recipients were treated with ferritin after 4 weeks. **B** IF staining of BMDNs after ferritin injection in bone marrow transplanted mice (MPO, green; CitH3, red; DAPI, blue. Scale bar = 40 μm). **C** Scanning electron microscope images of BMDNs (Scale bar = 10 μm). **D** Peripheral blood DNA concentration. **E** and **F** Peripheral blood and lung neutrophil frequency. **G**-**I** Serum inflammatory factors (IL-6, MCP-1 and TNF-α) levels. **J** and **K** Lung wet-to-dry ratios and lung injury scores. **L** and **M** HE staining and Masson staining images of lung tissue. **N** and **O** Inflammatory factor-associated mRNA levels in lung tissue

To further confirm the essential roles of PAD4, NE, and ROS in promoting ferritin-induced inflammatory responses, we constructed Padi4 −/−, Elane −/−, or Cybb −/− mice, respectively, and transplanted the bone marrow of the gene-edited mice as donors into the WT mice. Then we used ferritin injection to stimulate the receptor mice (Fig. 5A). As expected, BMDNs of mice transplanted with Padi4−/−, Elane−/−, or Cybb−/− BM showed lower levels of NET release (Fig. 5B-D). Meanwhile, we found that deletion of these genes resulted in a reduction in peripheral neutrophil frequency and hepatic neutrophil infiltration (Fig. 5E-F), inhibition of ferritin-stimulated up-regulation of serum cytokine levels (Fig. 5G-I), and a significant reduction in lung tissue damage (Fig. 5J, K). The results of HE staining and Masson staining of lung tissues showed that transplantation of Padi4−/−, Elane−/− or Cybb−/− BM could partially reverse ferritin injection-induced oedema, inflammatory infiltration and fibrosis in mouse lung tissue (Fig. 5L, M) mRNA levels of Il1b and Il6 were also downregulated (Fig. 5N, O). The above results suggest that ferritin promotes NET formation and inflammatory responses in a PAD4-, NE-, and ROS-dependent manner.

### MSR activation is critical for ferritin-induced neutrophil NETosis and acute lung injury

**Fig. 6:**
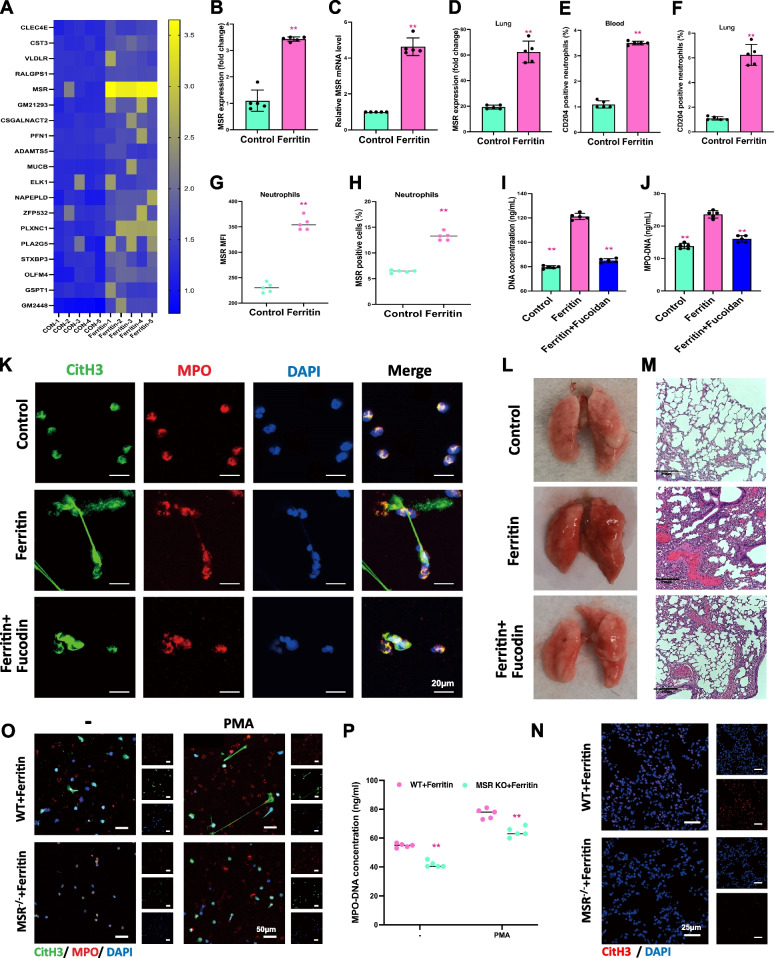
Ferritin promotes the production of NETs by activating Msr. **A** Heatmap of differentially expressed mRNA from BMDNs extracted for RNA-seq after ferritin stimulation applied to mice. **B** and **C** WB and qPCR results of Msr protein and mRNA levels in BMDNs. **D** Msr protein expression levels in lung tissues. **E** and **F** Proportion of neutrophils in peripheral blood and lungs of mice after ferritin stimulation. **G** and **H** Mean fluorescence intensity of MSR and percentage of Msr-positive cells in ferritin-treated human neutrophils. **I**-**J** DNA concentration and MPO-DNA concentration after ferritin injection using the Msr inhibitor Fucodin combined with ferritin. **K** IF images of BMDNs in mice after ferritin injection using the Msr inhibitor Fucodin combined with ferritin (MPO, red; CitH3, green; DAPI, blue. Scale bar = 20 μm). **L** and **M** Morphological and HE-stained images of lungs in mice. **N** IF images of CitH3 in lung tissue of WT and Msr ablated mice treated with ferritin (CitH3, red; DAPI, blue. Scale bar = 25 μm). **O** IF staining and (**P**) MPO-DNA complex concentration of BMDNs from ferritin-treated WT and Msr−/− mice with PMA stimulation in vitro

Next, we wanted to explore the specific molecular mechanisms underlying ferritin-induced neutrophil NETosis, leading to acute lung injury. We applied RNA-seq to BMDNs isolated from ferritin-injected mouse models (*n* = 5 vs. 5) to screen for key molecules in this process. We focused on molecules related to intrinsic immunity and ferritin receptors (Fig. 6A). The differential enrichment analysis revealed that the gene responsible for encoding macrophage scavenger receptor, MSR, was considerably upregulated. We conducted WB and PCR assays on BMDNs extracted from mice in the ferritin-treated and control groups. The results showed that ferritin stimulation induced upregulation of both MSR protein levels and mRNA levels in neutrophils (Fig. 6B-C). While examining mouse lung tissues, we found that injection of ferritin increased MSR protein expression simultaneously (Fig. 6D) we used flow cytometry to measure the proportion of CD204+ neutrophils in ferritin-treated mice’s peripheral blood and lung tissue. Results showed an upregulation of CD204+ neutrophils in both circulating and lung tissue (Fig. 6E-F). The same results were verified in neutrophils extracted from human peripheral blood, where ferritin treatment also induced upregulation of MSR expression in human neutrophils (Fig. 6G-H). Fucodin is an Msr antagonistic ligand. DNA concentration assay and MPO-DNA assay showed that in vitro application of fucodin significantly inhibited ferritin-induced neutrophil NETs production (Fig. 6I-J). This was confirmed by immunofluorescence results of NETs markers CitH3 and MPO (Fig. 6K). In vivo experiments found that ferritin-injected mice treated with Fucodin experienced significant relief from inflammatory infiltration and lung tissue injury (Fig. 6H-I). Additionally, we observed a decrease in the spontaneous formation of NETs by BMDNs from Msr−/− mice and a reduced ability to produce NETs in response to PMA (Fig. 6O). Ferritin promoted NETs production by neutrophils from WT mice but not Msr−/− mice (Fig. 6P). In addition, IHC results showed that CitH3 expression was significantly reduced in lung tissues of Msr−/− mice compared to WT mice after receiving ferritin injection (Fig. 6N). Taken together, these results suggest that Msr has a critical role in ferritin-induced NET formation.

### Msr ablation effectively alleviates ferritin-induced lung injury

**Fig. 7:**
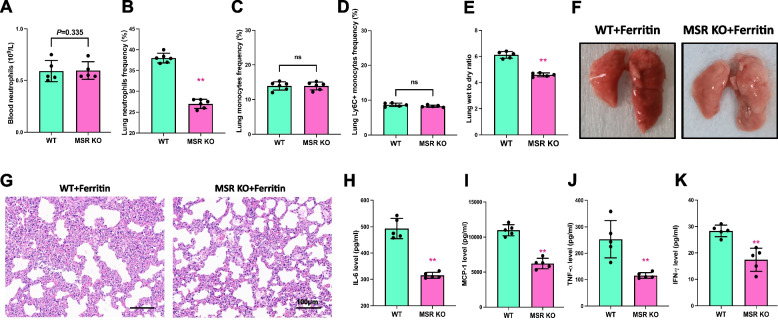
MSR ablation prevents systemic and pulmonary inflammation caused by ferritin. **A** and **B** Neutrophil frequency in peripheral blood and lung tissue of WT and MSR KO mice after ferritin treatment. **C** and **D** After ferritin treatment, lung tissue monocytes and Ly6C+ neutrophils frequency in WT and MSR KO mice. **E** Lung wet-to-dry ratio of WT and MSR KO mice with 12 h ferritin stimulation. **F** and **G** HE and morphological images of ferritin-injected WT and MSR KO mice. **H**, **I**, **J** and **K** After ferritin injection, serum levels of inflammatory factors including IL-6, MCP-1, TNF-α and IFN-γ in WT and MSR KO mice

Next, we investigated the role of Msr in ferritin-induced acute lung injury in mice. Our findings revealed that, while the number of neutrophils in the blood remained unaffected by Msr1 ablation (Fig. 7A), the lungs of Msr1 −/− mice treated with ferritin showed less neutrophil infiltration compared to WT mice (Fig. 7B). Moreover, we analyzed the proportion of monocytes and Ly6C+ cells in lung tissue and found no significant differences between the Msr ablation and WT groups (Fig. 7C, D). As shown in Fig. 7F and G, the degree of inflammatory infiltration was significantly reduced in the lung tissue of mice in the Msr ablation group following ferritin stimulation. Furthermore, the levels of inflammatory factors, including IL-6, MCP-1, TNF-α, and IFN-γ, were significantly downregulated in the lung tissue (Fig. 7H-K). Based on these findings, we concluded that Msr knockout could effectively alleviate ferritin-induced lung injury, further confirming the crucial role of Msr in the process of ferritin-induced lung injury.

### Elevated levels of ferritin promote NET formation in severe sepsis patient

**Fig. 8:**
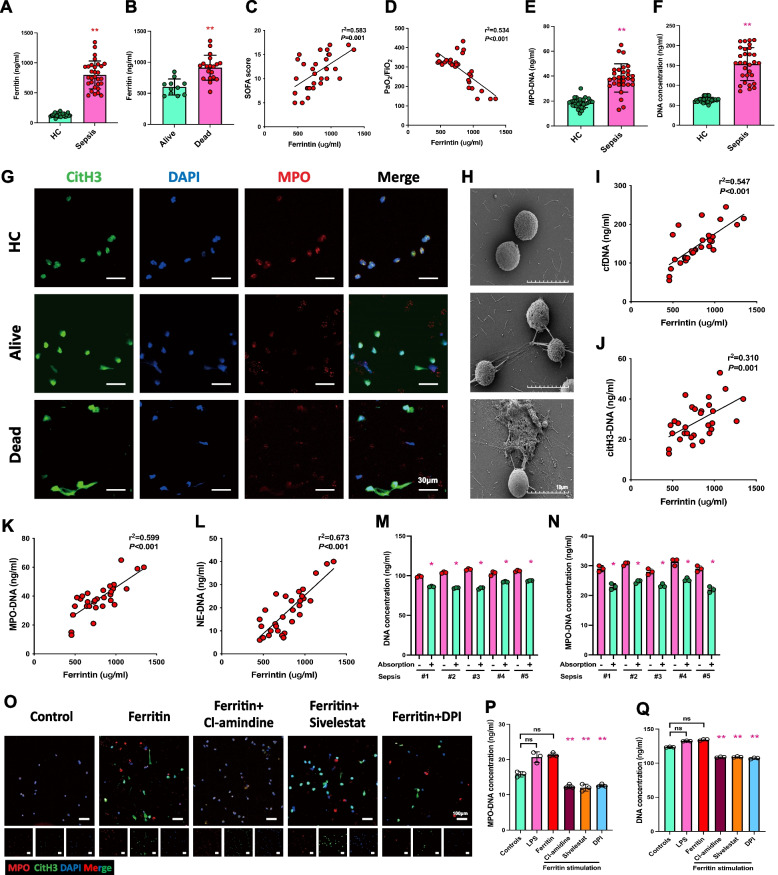
Up-regulation of ferritin in patients with sepsis leads to increased NETs production and exacerbates sepsis-associated acute lung injury. **A** Serum ferritin in healthy people and patients with sepsis (*n* = 30) and controls (*n* = 30). **B** Serum ferritin was detected in alive and dead sepsis patients. Alive (*n* = 11) and dead (*n* = 19). **C** and **D** Evaluate the correlation between serum ferritin concentration and the degree of lung damage (SOFA score and PaO2/FiO2) in sepsis patients. **E** and **F** MPO-DNA and DNA concentration in healthy people and patients with sepsis (*n* = 30) and controls (*n* = 30). **G** Representative NETosis images of alive-, dead-sepsis patients in comparison with healthy control. (MPO, red; CitH3, green; DAPI, blue. Scale bar = 30 μm). **H** SEM images depicting the release of NETs in septic or healthy neutrophils. (Scale bar = 10 μm). **I**, **J**, **K** and **L** The correlation between ferritin and NETs-related indicators (cfDNA, citH3-DNA, MPO-DNA and NE-DNA). **M** and **N** Stimulation of healthy donor neutrophils was performed using serum from five septic patients (serum ferritin absorbed and unabsorbed, respectively). Cell-free DNA and MPO-DNA complexes were quantified. **O**, **P** and **Q** Inhibition of ferritin-induced NET formation by PAD4, NE, ROS, and Msr1 inhibitors detected by IF of MPO (green), citH3 (red), and DAPI (blue) (Scale bar = 100 μm) in corresponding with MPO-DNA and cell-free DNA complexes

Sepsis is a severe inflammatory response syndrome in the clinic. Recent studies have shown that patients with sepsis have high levels of circulating ferritin, and the degree of ferritin elevation is strongly linked to their prognosis [19]. To measure the ferritin levels in patients with sepsis, we gathered blood samples from septic patients in clinical. The results showed that patients with sepsis had significantly higher ferritin levels in their bloodstream than healthy individuals (Fig. 8A). We also found that non-surviving patients showed a more significant increase in peripheral blood ferritin levels compared to surviving patients (Fig. 8B). Severe sepsis often leads to systemic multi-organ dysfunction, with sepsis-associated acute lung injury emerging as a significant contributor to unfavorable patient outcomes. We further analyzed the systemic conditions and lung injury in patients with clinical sepsis-related lung injury. The results showed that in patients with sepsis, peripheral blood ferritin levels were positively correlated with patients’ SOFA scores and negatively correlated with PaO2/FiO2 (Fig. 8C, D). Previous studies have shown that sepsis patients exhibit elevated circulating NETs. Consistent with our previous study, sepsis patients showed increased levels of dsDNA (Fig. 8E) and MPO-DNA complexes (Fig. 8F). Neutrophils from severe sepsis patients generate more NETs when stimulated with PMA, according to IF and SEM results (Fig. 8G-H). We have previously observed that ferritin promotes the production of NETs by neutrophils in a mouse model. Consistent with the results of the animal studies, NET-associated markers (including cfDNA, citH3-DNA, MPO-DNA, and NE-DNA) are positively associated with ferritin levels in clinical patients (Fig. 8E-H). To further determine the role of serum ferritin on neutrophil NETs production, we first extracted peripheral blood neutrophils from healthy control and stimulated the neutrophils using serum from patients with sepsis (pre- and post-absorption for ferritin). The ability of serum to promote NET formation was significantly attenuated after ferritin absorption (Fig. 8M, N). In Fig. 4, we demonstrated that ferritin-induced generation of NETs was dependent on PAD4, NE, and ROS. Herein, we stimulated neutrophils extracted from healthy controls using high-ferritin-containing serum from sepsis patients along with PAD4, NE, and ROS inhibitors. The IF and MPO-DNA results showed that inhibiting PAD4, NE, and ROS reduced NET production induced by high ferritin serum (Fig. 8O, P and Q). In conclusion, we demonstrated that elevated ferritin in patients with severe sepsis promotes the production of NETs and exacerbates septic lung injury. Inhibiting PAD4, NE, and ROS are effective in relieving high ferritin-induced NETs production.

## Discussion

Ferritin is an important protein complex responsible for storing iron in almost all organisms. It plays a crucial role in various metabolic pathways, inflammatory processes, stress responses, and in the pathogenesis of cancer and neurodegenerative diseases [20]. During disease, ferritin acts as an acute response phase protein involved in a variety of pathophysiological processes including inflammation, upper respiratory infections, or autoimmune diseases [21, 22]. Its synthesis is mediated by several proinflammatory factors such as interleukin-1β (IL-1β), IL -2, IL-6, and TNF-α, among other proinflammatory factors activated along the pathway of the transcription factor NF-κВ [23]. Researchers are currently focusing on hyperferritinemia and ferritin-induced inflammatory factor storms in light of the COVID-19 pandemic [24]. Evidence suggests that in addition to being activated for production by inflammatory factors, ferritin simultaneously can contribute to the inflammatory storm and ultimately exacerbate the progression of organ function impairment. A novel large-sample clinical study demonstrated the predictive value of serum ferritin in the short- and long-term prognosis of sepsis [25]. Past studies have shown a strong association between serum ferritin levels and acute lung injury. A transient iron-deficient diet in a rat model was effective in reducing the risk of acute lung injury [26]. While studies have indicated a connection between serum ferritin and sepsis-associated acute lung injury, the specific mechanisms involved have yet to be fully explored. In our study, we found hyper-ferritin could lead to inflammatory factor storms and induce acute lung injury.

NETs, a reticular substance released by neutrophils, were first identified by Brinkman et al. in 2004 [27]. It consists of chromatin wrapped in histones and abundant granular proteins that encapsulate MPO, NE, cathepsin G, and lactoferrin [28]. Numerous studies have shown that overproduction of NETs is associated with severe organ damage and poor prognosis in patients with clinical sepsis [29, 30]. Our past studies have focused on the role played by neutrophils in intrinsic immunity during sepsis-associated acute lung injury. We investigated the regulatory mechanisms of NETs and their involvement in sepsis-associated lung injury. NETs affect multiple pathways including ferroptosis [31], autophagy [32], and pyroptosis [14]. Meanwhile, recent studies have shown that multiple mechanisms are involved in the promotion of NETs release during inflammation, further exacerbating sepsis progression [15]. Herein, we first demonstrate that hyper-ferritin increases NET production during sepsis-associated lung injury. Our results showed that ferritin can accelerate the progression of acute lung injury by stimulating NET production.

Further, we investigated the potential molecular mechanisms by which ferritin induces NET formation. Previous studies have shown that NET generation requires the involvement of NE and PAD4 [33]. At the same time, some researchers have suggested that ROS are also essential in the formation of nets [34]. However, novel studies have shown that granulocyte-macrophage colony-stimulating factor (GM-CSF) and TNF can induce NETosis in a ROS-independent, but PAD4-dependent, manner [35]. These suggested that the mechanism of NET formation varies. Here we demonstrate through the bone marrow transplantation technique using inhibitors and gene-edited mice demonstrated that a ferritin-mediated increase in NETs production requires the combined involvement of NE, PAD4 and ROS.

Given that ferritin stimulates the formation of NETs in neutrophils, we have focused on ferritin receptor-associated proteins when investigating their specific mechanisms. Currently, studies on neutrophil-associated ferritin receptors are still limited. Our sequencing results and genetic findings suggest that the ferritin receptor MSR may be of value during ferritin-induced NET formation. MSR normally functions as a membrane-bound scavenger receptor on macrophages and monocytes during inflammation [16]. Recent studies suggest that MSR is simultaneously involved in the internalization of iron in the organism and maintaining iron homeostasis as a ferritin receptor [17]. In the mouse hepatitis model, MSR was shown to be involved in the activation of neutrophils and the generation of NETs [18]. The above results suggest that MSR may be crucial in ferritin-induced NET formation, a hypothesis we tested in the present article.

In this research, we utilized ferritin obtained from the equine spleen to stimulate a mouse model through intraperitoneal injection. Since the equine spleen is a common primary source of ferritin for ferritin-related mechanisms, we did not use ferritin derived from humans or mice directly in our isolated neutrophil or in vitro mouse models [36]. We acknowledge that our article has limitations, as it is unclear whether this model accurately mimics the hyperserotonemia state of patients with sepsis, despite previous relevant studies. Additionally, we performed RNA-seq to validate the involvement of MSR in ferritin-triggered neutrophil activation and enhanced NET formation. Our findings suggest that ferritin induces an increase in MSR expression both on the cell surface and throughout the cell. Moreover, combined MSR knockdown significantly inhibited ferritin-induced acute lung injury in a mouse model. However the specific mechanism of ferritin-induced MSR upregulation requires further exploration. Identifying this mechanism could provide innovative treatments for reducing lung injury caused by increased NETs due to ferritin-induced MSR upregulation.

In conclusion, we demonstrated that hyper-ferritin can induce systemic inflammation and increase NET formation in an MSR-dependent manner. This process relies on PAD4, NE, and ROS, further aggravating acute lung injury. In the clinic, high serum ferritin levels are associated with elevated NETs and worse lung injury, which suggests a poor prognosis for patients with sepsis. Our study indicated that targeting NETs or MSR could be a potential treatment to alleviate lung damage and systemic inflammation during sepsis.

### Supplementary Information

**Additional file 1.**

## Data Availability

All data generated or analyzed during this study are included in this published article. Data will be made available on request.
