# Supplementary figures and images for: Ferritin-mediated neutrophil extracellular traps formation and cytokine storm via macrophage scavenger receptor in sepsis-associated lung injury
Source: Cell Commun Signal. 2024 Feb 2;22:97. doi: 10.1186/s12964-023-01440-6 (PMC10837893; doi:10.1186/s12964-023-01440-6)

## Slide 1
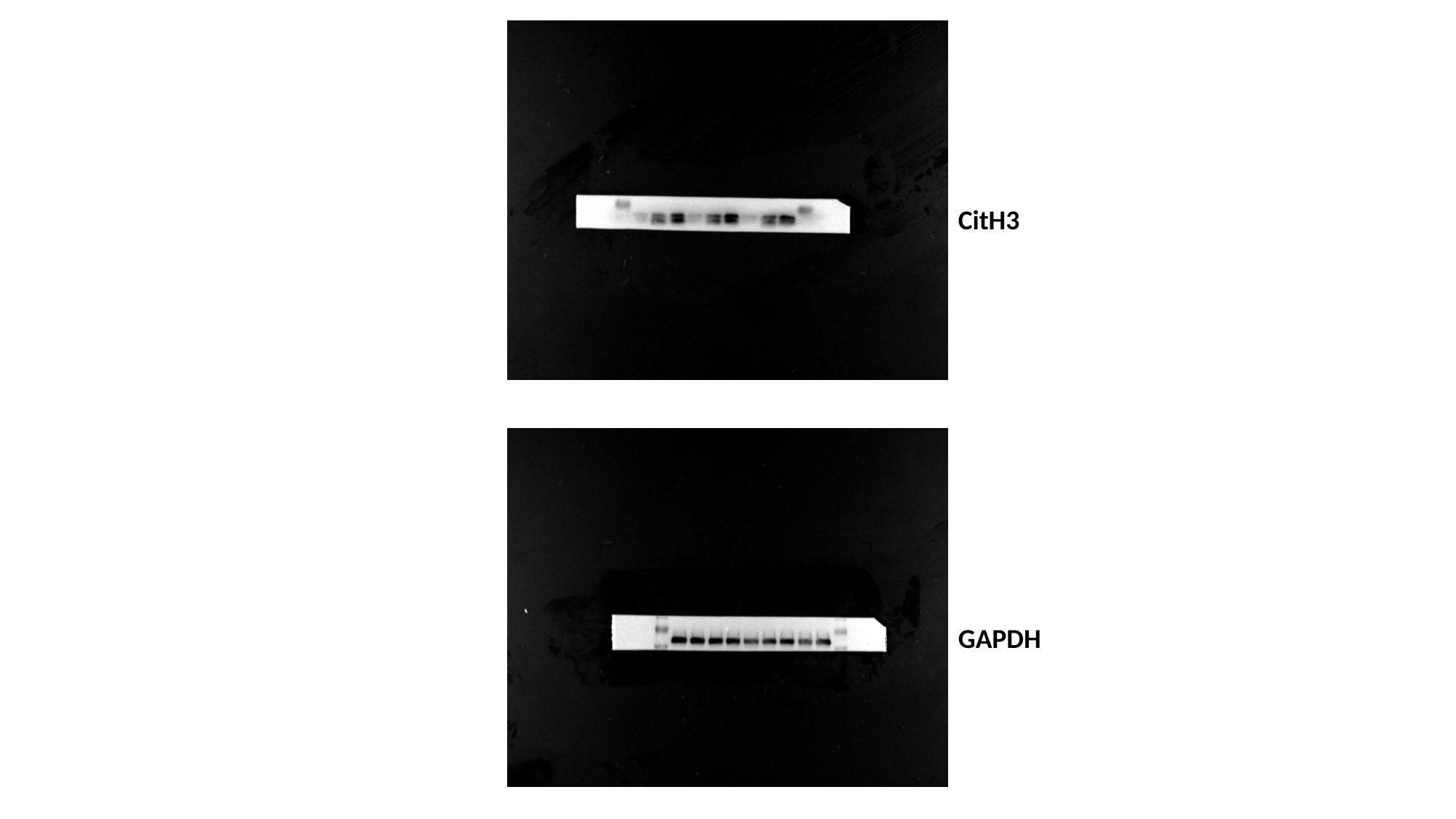

CitH3
GAPDH

Supplement: Supplementary file 2 — Additional file 1. [file 12964_2023_1440_MOESM1_ESM.pptx]
